# Extension of the Thermographic Signal Reconstruction Technique for an Automated Segmentation and Depth Estimation of Subsurface Defects

**DOI:** 10.3390/jimaging6090096

**Published:** 2020-09-11

**Authors:** Alexander Schager, Gerald Zauner, Günther Mayr, Peter Burgholzer

**Affiliations:** 1 Josef Ressel Centre for Thermal NDE of Composites, University of Applied Sciences Upper Austria, 4600 Wels, Austria; alexander.thomas.schager@gmail.com (A.S.); gerald.zauner@fh-wels.at (G.Z.); 2 RECENDT—Research Centre for Nondestructive Testing, 4040 Linz, Austria; peter.burgholzer@recendt.at

**Keywords:** active thermography, pulse thermography, thermographic signal reconstruction, depth estimation, automatic defect segmentation, defect imaging, composite material

## Abstract

With increased use of light-weight materials with low factors of safety, non-destructive testing becomes increasingly important. Thanks to the advancement of infrared camera technology, pulse thermography is a cost efficient way to detect subsurface defects non-destructively. However, currently available evaluation algorithms have either a high computational cost or show poor performance if any geometry other than the most simple kind is surveyed. We present an extension of the thermographic signal reconstruction technique which can automatically segment and image defects from sound areas, while also estimating the defect depth, all with low computational cost. We verified our algorithm using real world measurements and compare our results to standard active thermography algorithms with similar computational complexity. We found that our algorithm can detect defects more accurately, especially when more complex geometries are examined.

## 1. Introduction

In modern manufacturing, lightweight materials are often used to reduce costs over the lifetimes of the products. For this reason, both the automotive industry and the aviation industry, amongst others, are increasingly using fiber reinforced polymers (FRPs). FRPs are manufactured by fusing together prefabricated sheets, which already include the fibers. Thus, FRPs tend to develop interlaminar defects, which occur when air or foreign materials, e.g., foils, are embedded between two layers. Although the thickness of the interlaminar defects is often only a fraction of the prefrabricated sheet thickness, they structurally weaken FRPs. For this reason, FRP components are either developed with a considerable safety margin or are tested non-destructively.

Three physical principles are mainly used to detect interlaminar defects of FRPs non-destructively. The most accurate is 3D X-ray computed tomography (3D-XCT), but there are several drawbacks to this method which have to be considered. 3D-XCT requires expensive equipment, and depending on the voxel size, requires considerable measurement and evaluation times. Another disadvantage is the very limited sample size and the use of ionizing radiation. The aviation industry predominantly uses ultrasonic testing (UT), where an ultrasonic probe scans the component in a grid-like manner. The drawbacks of this method are that UT is not contactless and the large signal acquisition time that results from scanning.

An emerging method is active thermography (AT). In AT, components are heated or cooled while the temperature change is observed by an infrared (IR) camera. Thus, the measured raw data can be viewed as an IR video. Modern megapixel IR cameras are able to record large surface areas quickly without contact and with relatively inexpensive equipment. Due to the short signal acquisition time, it is possible to use AT for in-line testing of injection molded components. However, the evaluation algorithm for such components quickly becomes a time bottleneck in real production environments. Ideally, the algorithm should segment the defective from non-defective areas and at the same time determine the depths of the defects within a reasonable computational time.

An AT measurement begins with the heating or cooling of a specimen relative to the ambient temperature to create a temperature gradient called excitation. For most applications only heating is used. Defects that inhibit or accelerate the local heat diffusion can be identified by analyzing the measured thermal sequence. However, the diffusion characteristics of the so-called "thermal waves" [1] limit the depth resolution [2]. Defects that lie deeper below the surface can only be detected with a lower spatial resolution. The different types of AT can be subdivided by the type of excitation; however, the fastest signal acquisition is achieved by so-called pulse thermography (PT). Here, the surface of a specimen is optically excited with a Dirac-delta-like heat pulse at time t=0, which is usually provided by high-powered flash lamps or lasers. This type of excitation is most suitable for in-line testing in an industrial environment where the signal acquisition time should be kept as short as possible. After the initial heat pulse, the cooling of the surface of the specimen is recorded with an IR camera until the steady-state, i.e., ambient temperature, is reached. Two measurement setups are used in PT: the transmission and reflection setups. In the transmission setup, the specimen is positioned between the IR camera and the excitation source. This means that both sides of a specimen need to be accessible in order to record a measurement, which is often not possible under industrial conditions. In the reflection setup, the excitation plane of the specimen is also the measurement plane. Therefore, only one side of the specimen needs to be accessible for the measurement. In this work PT is only investigated in the reflection setup.

There are a large number of post-processing algorithms for PT measurements in reflection. Most of these algorithms use the time-temperature sequences to gain insight into the material; i.e., they observe how the temperature of a pixel changes over time. Since there are thorough literature reviews [3,4], we only give a brief introduction to the most important algorithms.

One of the first algorithms developed was pulse phase thermography (PPT) [5,6]. In PPT, the 1D Fourier transform and then the signal phase are calculated from each time-temperature sequence of the thermal video. Defects can be identified by differences in the phase values. Usually only the image with the highest contrast is used for defect detection. It is possible to correlate the phase and frequency information with a defect depth using the blind-frequency method [7,8]. Another widely used evaluation algorithm for PT data is the principal component thermography [9], wherein the measurement data are analyzed using the singular value decomposition. More recently, this approach was used for the analysis of long pulse thermography [10,11], wherein due to increased excitation time deeper defects can be imaged at the cost of a longer signal acquisition time.

Another method is the thermographic signal reconstruction (TSR) algorithm [12,13]. Here the time-dependent temperature values are plotted on a double-logarithmic scale. Each logarithmic time-temperature sequence is then approximated by a polynomial. TSR has triggered many other algorithms, such as improvements to the early time method [14] and the RGB projection technique [15]. In [16] the absolute peak slope time method (APST) was presented. Here the time-temperature sequences are multiplied by $t^{n}$ with *n* being a constant between 0.4 and 0.5. Then a non-linear fit is applied to these signals, based on a simplified mathematical model derived by [17]. With this method it is possible to estimate the defect depth. A similar strategy was pursued in [18], the reference free dynamic thermal tomography (DDT). In DDT, time-temperature sequences are multiplied by $t^{n}$ with *n* being between 0.3 and 0.43. It is postulated that the “arg min” of these multiplied time-temperature sequences can be used directly to estimate a defect depth.

Another image reconstruction method was recently proposed by [19], the virtual wave concept (VWC). Here, each time-temperature sequence is first mathematically transformed into a virtual ultrasonic wave. Ultrasonic reconstruction algorithms are then used to estimate defect depths [20,21].

All of these algorithms have their strengths and weaknesses. For certain samples, e.g., PPT can be used to visualize defects quickly. TSR is one of the most commonly used algorithms for estimating the effective thermal diffusivity of samples. Furthermore, the defect depths can also be roughly estimated with the RGB projection technique. On the other hand, more advanced methods such as the VWC can be used to produce detailed defect maps. In addition, VWC can deal with structured excitation [22], enabling defect resolutions which cannot be achieved with PT. Algorithms such as PPT and TSR have short computing times, but have difficulties with more complex sample geometries, such as varying sample thicknesses. Algorithms suitable for such samples, e.g., APST and VWC, generally have longer computing times.

We propose an algorithm which can produce reasonably accurate defect depth maps, even for complex geometries, while keeping the computational cost low. This is done by reinterpreting the technique from [18] with knowledge from TSR. In this way we can segment defective from non-defective (sound) areas and give an estimate of the defect depth.

This publication is structured as follows. First, a theoretical section is provided that explains the signal format and measurement acquisition, along with the differential equations which describe heat transfer in solids (heat equation). We also show numerical solutions of the heat equation, which form the basis of the TSR method. The second section describes our algorithm in detail and uses the simulation results from the previous section to link to the TSR method. In the last section, we validate our algorithm using real samples from a pre-production run where ground truths are provided by 3D-XCT scans. We also compare our results with those obtained using standard methods, such as TSR and PPT.

## 2. Thermographic Evaluation Method

### 2.1. Signal Acquisition

**Figure 1:**
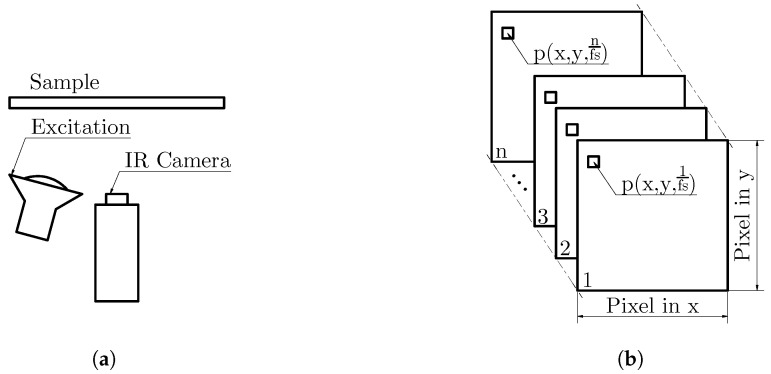
Pulsed thermography experiment: (**a**) measurement setup in reflection and the (**b**) data format of a thermographic measurement.

A schematic of a PT measurement in reflection setup is shown in Figure 1a. The image shows a sample, a flash lamp and the IR camera. Often more than one flash lamp is used to achieve a spatially homogeneous excitation of the specimen surface. In Figure 1b the data format of a thermographic measurement is shown. A PT measurement can be thought of as an IR video with *n* frames, recorded at a sampling frequency, also called frame rate, $f_{s}$. Since both the IR camera and the sample are stationary, it is useful to analyze the temporal evolution of all pixels, the so-called temperature–time sequence p(x, y, t).

### 2.2. Heat Diffusion Equation

Heat transfer in solids via conduction is described by a partial differential equation, for the temperature T(x, y, z, t) as a function of space and time—the heat diffusion equation:

(1) $$\frac{\partial T}{\partial t}-\left(\alpha_{x}\left(x\right)\frac{\partial^{2}T}{\partial x^{2}}+\alpha_{y}\left(y\right)\frac{\partial^{2}T}{\partial y^{2}}+\alpha_{z}\left(z\right)\frac{\partial^{2}T}{\partial z^{2}}\right)=0.$$

Given the geometry of a body, its boundary conditions and the initial condition, the temperature of a body can be calculated at any point in space at any time. The boundary condition describes how a body interacts with its surroundings. The initial condition is simply the temperature of the body at time t=0, $T_{0}\left(x,y,z,0\right)$, for example, the ambient temperature $T_{a}$. The thermal diffusivity, α(m2s), describes ℌhow fastℍ temperature diffuses through a body. The thermal diffusivity relates material intrinsic parameters in the following way:

(2) $$\alpha =\frac{k}{\rho \;c}.$$

Here k(W(m·K)) denotes the thermal conductivity, which describes how well heat energy can be transported through a body, while ρ(kgm3) denotes the density. c(J(kg·K)) refers to the specific heat capacity, which describes how the energy relates to temperature. Analytic solutions of Equation (1) only exist for special cases. In PT for example, the excitation can be modeled by a Dirac-delta-like homogeneous heat impulse on the surface z=0 at time t=0. For a semi-infinite homogeneous 1D slab, the slab starts at z=0 and continues to infinity. Solving the heat equation yields [23]:

(3) $$T\left(z,t\right)=\frac{Q_{0}}{\sqrt{t\pi }\zeta }\exp\left(-\frac{z^{2}}{4\alpha_{z}t}\right)+T_{a}.$$

Here, $\zeta =\sqrt{ck\rho }$ denotes the thermal effusivity, which describes how well heat energy can be coupled into materials. The total energy of the excitation is described by $Q_{0}$, while $T_{a}$ is the ambient temperature. In the reflection setup only the surface temperature of the slab is measured. Setting z=0 causes the exponential term to become equal to one. With the exception of carefully controlled experiments, with which the thermal effusivity can be estimated, the main takeaway is that the temperature decays proportionally to $T\left(t\right)\propto t^{-\frac{1}{2}}$, if the ambient temperature is subtracted. This is well known and the reason why temperature–time diagrams for pulse thermography are often depicted in log–log scales.

However, non-destructive evaluation (NDE) in active thermography aims to solve the inverse problem, meaning to detect internal boundaries, i.e., defects, for a known excitation and a measured surface temperature distribution. For this problem we cannot directly use Equation (1), with which the temperature distribution of a known geometry and excitation can be calculated. However, Equation (1) can be used to show the trend of the surface temperature for a specific geometry subject to a PT excitation and to simulate noise free data for the validation of the proposed thermographic evaluation method.

### 2.3. Numerical Solution of the Heat Equation

**Figure 2:**
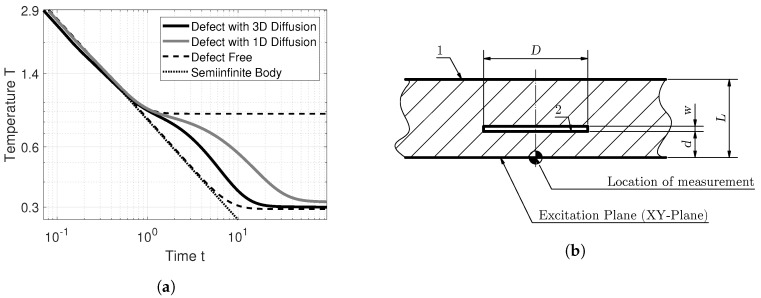
Numerical solution of the heat equation: (**a**) simulated temperature–time sequences and (**b**) the geometry used for the numerical simulations.

COMSOL Multiphysics^®^ can be used to simulate several temperature–time diagrams (Figure 2a), which were obtained by using the geometry depicted in Figure 2b, assuming adiabatic boundary conditions; i.e., the sample is thermally isolated from its environment. The temperature was measured at the surface, above the center of an air pocket, marked in Figure 2b. The geometry is made up of two different materials, material 1 the composite material, and material 2, an air pocket, which simulates a delamination. The physical parameters used are shown in Table 1. Here material 1 has an anisotropic diffusion character, which is typical for FRPs. The in-plane thermal conductivity $k_{xy}$ was three times larger than the through-plane thermal conductivity $k_{z}$. Equation (2) shows that the thermal conductivity impacts the thermal diffusivity linearly.

Figure 2a shows four temperature–time sequences. The temperature–time sequence of Equation (3), the semi-infinite body, shows an expected slope of −12, which holds when $T_{a}=0$. The defect-free (sound) temperature curves show the temperature–time sequences if no defect is present. The upper dashed line (- -) represents the temperature evolution resulting from a slab with a thickness of *d*, while the lower line (- -) is the result of a slab with a thickness of *L*. The upper line shows the time sequence of a so called “flat-bottomed hole” (FBH), which are extensively used in active thermography research due to their ease of manufacturing. However, it must be stressed that a temperature–time sequence produced by a FBH in and of itself does not indicate a defect. These temperature–time sequences were calculated using the analytic expression derived by [17]. The temperature curve of defect with 1D diffusion shows the numerical solution of the geometry depicted in Figure 2b, if only through-plane diffusion is permitted, i.e., one dimensional heat diffusion. This result was computed using the physical parameters of Table 1, but setting $k_{xy}=0$ for both material 1 and material 2.

We can interpret the temperature–time sequences of Figure 2a in the following way. Comparing the defect-free curves, we can see that the transition from the semi-infinite body to the adiabatic temperature plateau is self similar to a log–log plot. By comparing the defect-free behavior for the thinner body to the defect with 3D diffusion, we can see that an air pocket behaves similarly to the adiabatic plateau, albeit less pronounced. Looking at the defect with 1D diffusion, it is apparent that the temperature does not decay as fast as in the semi-infinite body; i.e., the slope of the temperature–time sequence is greater than −12. These types of temperature–time sequences are typical for large defect diameters *D* relative to the defect depth *d*. After an initial temperature build-up, the defect with 3D diffusion shows a faster temperature decrease than in the semi-infinite body, i.e., with a gradient of less than −12. This is expected because the heat above the defect diffuses laterally, which manifests itself in a faster decrease of temperature above the defect. If the slab thickness *L* were set to infinity, we would expect the defect with 3D diffusion to approach the semi-infinite body asymptotically. This means that the estimation of the through-plane thermal diffusivity $\alpha_{z}$ is less impacted by defects close to the excitation, especially if a large anisotropy regarding the thermal diffusivity is situated. Generally speaking, a large thermal anisotropy aggravates the detection of defects because less temperature buildup occurs compared to samples with small thermal anisotropy.

### 2.4. Thermograpic Signal Reconstruction (TSR) Algorithm

TSR is a method of estimating the through-plane thermal diffusivity $\alpha_{z}$. This estimate of $\alpha_{z}$ is referred to as $\alpha_{ez}$, the effective through-plane thermal diffusivity. The TSR algorithm can be described in the following way. First, the ambient temperature $T_{a}$ is subtracted from the temperature–time sequence, yielding ΔT. Then, a least squares polynomial regression is carried out in log–log scales. This polynomial is referred to as the TSR polynomial. Suggestions based on analytical studies [24] have been made, to determine the degree *n* of the TSR polynomial that should ideally be used. The optimal degree *n* depends on the sample under consideration and measurement acquisition parameters, but it is in the range of n=6 to n=9:

(4) $$\log\left(\Delta T\right)\approx \beta_{0}+\beta_{1}\log\left(t\right)+\beta_{2}\log{\left(t\right)}^{2}+\ldots +\beta_{n}\log{\left(t\right)}^{n},$$

where $\beta_{0\cdots n}$ are the polynominal coefficients. Then, the second analytic derivative of the TSR polynomial is calculated. The time $t^{*}$ at which the maximum of the second logarithmic derivative with respect to the logarithmic time occurs, connects the effective through-plane thermal diffusivity $\alpha_{ez}$ to the sample thickness *L* according to [13]:

(5) $$t^{*}=\arg\;\max\left(\frac{d^{2}\log(\Delta T)}{d{\left(\log\left(t\right)\right)}^{2}}\right)=\frac{L^{2}}{\alpha_{ez}\pi }.$$

If the slab thickness *L* is known, then the effective through-plane thermal diffusivity $\alpha_{ez}$ can be calculated. If the effective thermal diffusivity $\alpha_{ez}$ is lower than the expected thermal diffusivity $\alpha_{z}$ then something inhibits the heat flow, which could indicate a defect. However, it is also possible that a higher or lower $\alpha_{ez}$ is caused by a local variation of thickness.

The maximum of the second derivative can be calculated in two ways: either by evaluating the second analytic derivative, or by deriving the TSR polynomial three times, and finding and evaluating all real valued roots. The second derivative is in essence the curvature of the temperature–time sequences, and looking at Figure 2a, one would also expect to find the depth of the air pocket by the TSR method. In practice this is not the case, because polynomials can only approximate the temperature–time sequences. The sequence obtained by plotting the second derivative of the TSR polynomial often shows multiple peaks, wherein it is often not clear which were caused by an air pocket and which were due to oscillations (Runge’s phenomenon) of the polynomial.

## 3. Modification of Thermograpic Signal Reconstruction Method

**Figure 3:**
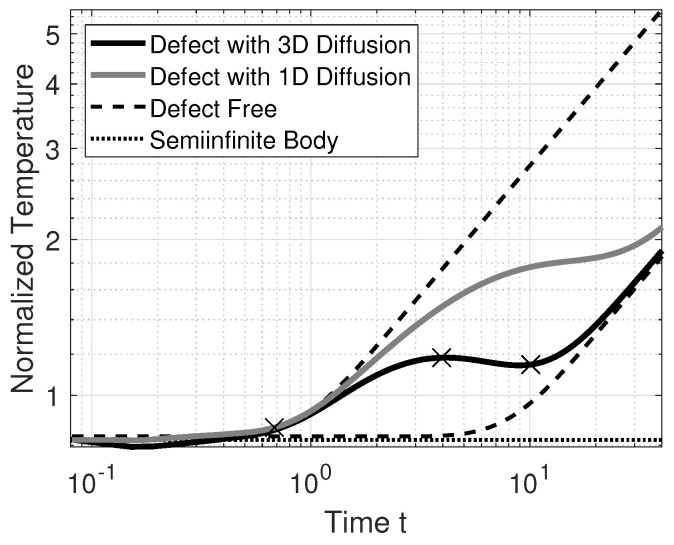
Normalized temperature–time sequences using simulation data multiplied by $\sqrt{t}$ (see text).

The basis of the proposed modification of TSR stems from the idea that there is more information contained in temperature–time sequences than merely $t^{*}$. The idea is to characterize a defect not indirectly by the effective thermal diffusivity, but by identifying a defective temperature field by the deviation from a sound temperature field using Equation (3). As an intermediate step, we multiply the temperature–time sequences from Figure 2a with $\sqrt{t}$. The result is depicted in Figure 3. Temperature–time sequences multiplied by $\sqrt{t}$ are herein referred to as “normalized temperatures.” A similar method was explored by [16], although the underlying ideas are different.

Figure 3 shows that temperature sequences which are affected by defects can be characterized by three characteristic times, labeled in the plot with crosses. The first cross indicates the time at which a temperature profile caused by a defect deviates from the defect-free temperature profile. The second cross shows the time at which the largest difference between a defective and sound region temperature profile, in a total least squares sense, occurs. The last cross indicates the time at which the cooling effect caused by 3D heat diffusion vanishes. This cross only occurs if adiabatic boundary conditions apply. The second and third cross are extreme values of the normalized temperature. The first cross can be made to occur at an extreme value of the normalized temperature. For example, this can happen if the ambient temperature was badly estimated, which resulted in an erroneous ΔT. However, we propose to let the time axis start at t0=2fs instead of t0=1fs, which has the added benefit of reducing oscillations in the TSR polynomial.

In contrast to [18], where only the arg min of the normalized temperature is evaluated, our proposed method of labeling defects is based on an analysis of whether the three distinct times occur in a normalized temperature–time sequence. Therefore, this method can be used when the thickness *L* of a sample varies locally. This also means that an FBH cannot be identified by this method, since it does not produce temperature–time sequences which would be typical for interlaminar defects. As the characteristic times are all located at extreme values of the normalized temperature, they can be found by curve sketching. If we fit a polynomial to the normalized temperature and derive it once, the real valued roots of this polynomial yield the characteristic times. Reducing the number of derivatives compared to TSR is desirable because derivation is an ill-posed operation.

### 3.1. Interpreting the Characteristic Times

**Figure 4:**
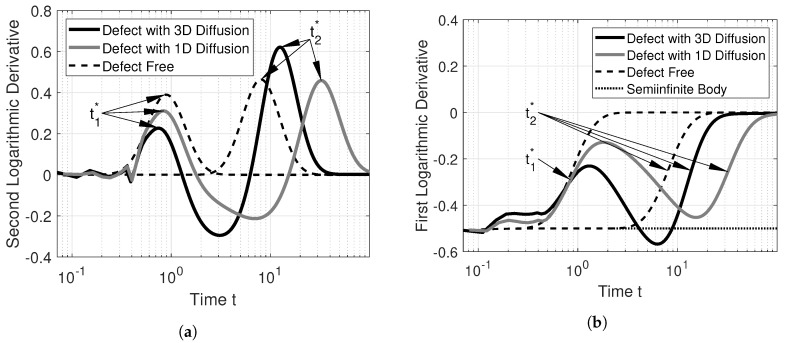
Interpreting the characteristic times: (**a**) second logarithmic derivative and (**b**) the first logarithmic derivative of simulation data.

To interpret the meaning of the characteristic times at the crosses from Figure 3, we calculate the logarithmic derivatives of its temperature–time profiles. The derivatives are calculated directly using the simulated data to remove any distortions which would be produced by first fitting the data with a polynomial. The second logarithmic derivative of the temperature–time sequences of Figure 2a is depicted in Figure 4a, while the first logarithmic derivative is depicted in Figure 4b.

We already established that a temperature–time sequence which is typical for a defect behaves similarly to the temperature–time sequence of an adiabatic boundary, and should in principle show up in the second logarithmic derivative. Furthermore, the times at which peaks in the second logarithmic derivative occur, $t_{1}^{*}$ and $t_{2}^{*}$, fulfill the equality from Equation (5).

Looking at the second logarithmic derivatives, Figure 4a, we see the first few values of the second logarithmic derivative of the defect with 3D diffusion and those with 1D diffusion fluctuate. This is caused by simulation errors. As the defect-free curves are calculated using an analytic expression, they do not suffer from these errors. We see that the second logarithmic derivative for the defect-free calculation looks remarkably similar to a Gaussian, which was also observed by [13]. However, this does not apply to real temperature–time curves, as the defects with 3D diffusion and that with 1D diffusion show. The first peak at $t_{1}^{*}$ is slightly earlier for 3D diffusion than for 1D diffusion. The peak itself is significantly dampened for the defect with 3D diffusion, which inhibits the detectability of interlaminar defects compared to FBHs.

Figure 4b shows the first logarithmic derivative. The *y*-axis of this figure starts at −0.5, which is caused by the term 1t in Equation (3). We find the times $t_{1}^{*}$ and $t_{2}^{*}$ at approximately the half-rise times of the first logarithmic derivative. Multiplying the temperature–time sequences, for example, by $\sqrt{t}$, does slide the first logarithmic derivative up by 0.5. By sliding up the signals from Figure 4b by 0.5, we see that the defect with 3D diffusion intersects with the *x*-axis two times, as indicated by the crosses in Figure 3. We can also see that the same is not true for defects with 1D diffusion, which do not intersect with the *x*-axis. Defects which behave according to the 1D heat diffusion cannot be detected by simply multiplying by $\sqrt{t}$. The main problem is in finding a value *m* by which the first logarithmic derivative should be slided up (or down), such that temperature–time sequences which indicate defects exhibit three zero crossings and such that the first zero crossing is near the half-rise time. For the experimental part we chose a constant of m=0.48, which was found experimentally.

### 3.2. Implementation of the Modification

Measurements are always subject to signal noise. Assuming that the standard deviation of the measurements is constant, after multiplying the temperature–time sequences by $t^{m}$ the standard deviation for the measurement at time $t_{i}$ is amplified by $t_{i}^{m}$. A better solution is to use the TSR polynomial from Equation (4). It can be easily verified that the addition of the value *m* to the polynomial coefficient $\beta_{1}$ results in equivalent temperature–time sequences by direct multiplication with $t^{m}$. Our algorithm can therefore be summarized as follows:

1. Fit a polynomial of the form of Equation (4) to the temperature–time sequence, but use t+1fs for the *x*-axis for improved stability of the polynomial.
2. Add the value 0.48 to the polynomial coefficient $\beta_{1}$.
3. Calculate the first derivative of this polynomial.
4. Evaluate the real valued roots of this polynomial and discard roots caused by oscillation (i.e., real valued roots outside the measured time).
5. If three or more roots are found this indicates a defect, and location of the first root roughly indicates the defect depth according to Equation (5).

## 4. Experimental Study

**Figure 5:**
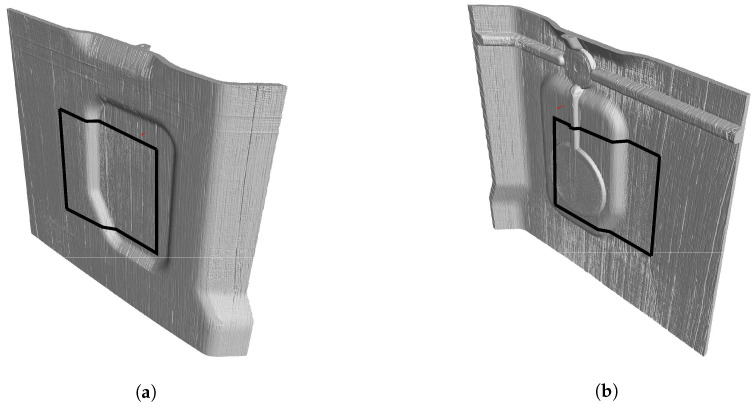
3D view of the test specimen: (**a**) front and (**b**) rear side. The voxel size of the measurement was 80 μm; for the area indicated by the black rectangle a voxel size of 30 μm was available.

We evaluate the performance of our modification of TSR by examining a sample from a pre-production run. This sample includes back molding for added structural integrity. The basic layer which is 2 mm thick consists of glass fiber reinforced plastic (GFRP), while the backmolding is made up of thermoplastics. Any defects in this sample can be attributed to the manufacturing process, which was not yet optimized for this sample. A basic assumption regarding the defects was obtained by a 3D-XCT scan. The capabilities of our algorithm were analyzed using this ground truth. The front side of the sample is depicted in Figure 5a and the rear side is depicted in Figure 5b. For this sample, the resolution of the 3D-XCT was set to 80μm, which proved to be too coarse for this sample. The black rectangle, projected on the surface, indicates a region for which the resolution was set to 30μm.

### 4.1. Measurement and Computational Complexity

We excited the sample from Figure 5a with two high powered flash lamps, each releasing approximately 1 kJ of radiative energy. The integration time of the IR camera was set to 1 m s and the frame rate was $f_{s}=25\;\mathrm{Hz}$. We recorded the surface temperature for 50 s at a spatial resolution of (912 × 1272) pixels. As the analog digital converter of our IR camera has a resolution of 14 bit, the measurement data have a size of 2.5 GB. Calculating the polynomial fit for each of the (912 × 1272) temperature–time sequences boils down to matrix multiplication. The so-called pseudoinverse, which is needed for the calculation of the polynomial coefficients, does not change and has to be calculated only once for the whole measurement. In fact the bottleneck of the algorithm is in calculating the roots of each polynomial, which can be formulated as an eigenvalue problem of a (n−1)×(n−1) matrix, with *n* being the degree of the polynomial. The computational complexity of our algorithm is therefore $O({\left(n-1\right)}^{3}\;N)$, where *N* is the number of temperature–time sequences. It must be noted that the algorithm is highly parallelizable, since each temperature–time sequence is analyzed independently. It must be noted that the computational complexity of most PT evaluation algorithms grows linearly with the number of temperature–time sequences *N*, because the measurement is analyzed on a pixel by pixel basis. However, most algorithms which enable defect depth estimation rely on computationally complex regression techniques such as non-linear fitting for APST and ADMM regularization for VWC. The computational convergence of these algorithms cannot be easily quantified as they operate iteratively and stop according to a user defined stopping criterion.

### 4.2. Results

**Figure 6:**
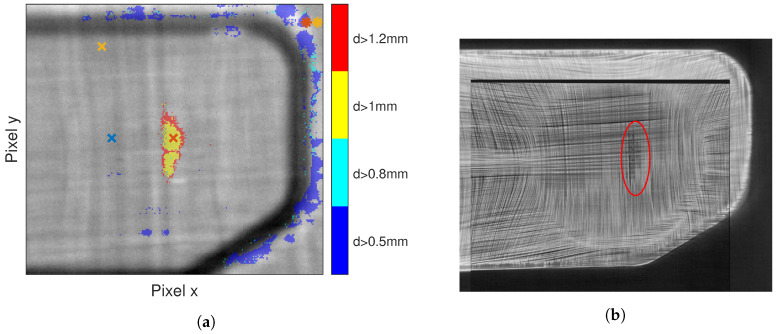
Comparison of active thermography with 3D-computed tomography: (**a**) results of the proposed algorithm and (**b**) the density map obtained by 3D-XCT at a depth of 1 mm from the front side.

To link the diffusion time at which the defect occurs to an actual defect depth, the through plane thermal diffusivity $\alpha_{z}$ has to be known. We estimated $\alpha_{z}$ using the TSR method. For the estimation of $\alpha_{z}$ we only considered a defect-free region, where no backmolding was added. We evaluated the peak second derivative for each temperature–time sequence in this region and calculated the mean of the results after removing outliers. Using Equation (5) and the known sample thickness at that region, we reached an effective thermal diffusivity of αez=2.310−7(m2s). The defect depths were measured from the front surface. The defect depth according to our modification to TSR is depicted in Figure 6a, and a sectional view of the 3D-XCT measurement at a defect depth of 1 mm is shown in Figure 6b.

For the results depicted in Figure 6a we used a polynomial of degree n=9 and added the constant m=0.48 to the polynomial coefficient $\beta_{1}$. The background image of Figure 6a is an IR image at time t=0.4 s. We then superimposed the results of our algorithm on the background image. We scaled the calculated depth map in the following way: the dark blue color indicates defects at depths between 0.5mm and 0.8mm, the light blue color indicates defects between 0.8mm and 1mm, the yellow color indicates defects between 1mm and 1.2mm and the red color shows any defects deeper than 1.2mm. We should emphasize here that this result was derived automatically, i.e., no user intervention was necessary, which makes this approach particularly interesting for industrial applications, e.g., in an automated production environment.

Figure 6b shows a sectional view of the 3D-XCT measurement at a depth of 1 mm. Darker regions indicate regions of low density, while bright regions indicate high density. We can see that the glass fibers are resolved individually and that they have a higher density than the thermoplastic matrix they are embedded in. Furthermore, we can spot an air inclusion, or other imperfection, indicated by the red ellipse. Comparing the shape of the defect in Figure 6b with our prediction from Figure 6a we note that identification of the size and depth of this defect is possible with good accuracy.

However, we were unable to verify shallower defects, marked in dark blue in Figure 6a. The reason could be that the resolution of 80μm, which was available for this region, is too coarse to resolve the defects, especially around curved surfaces. In general, defects close to the excitation plane are identified more easily with PT. In PT, unlike 3D-XCT or UT, we cannot directly estimate the through plane thickness *w* of defects; see Figure 2b. It is possible that for the blue regions *w* is so small that an extremely fine resolution of the 3D-XCT is required, regardless of surface curvature.

**Figure 7:**
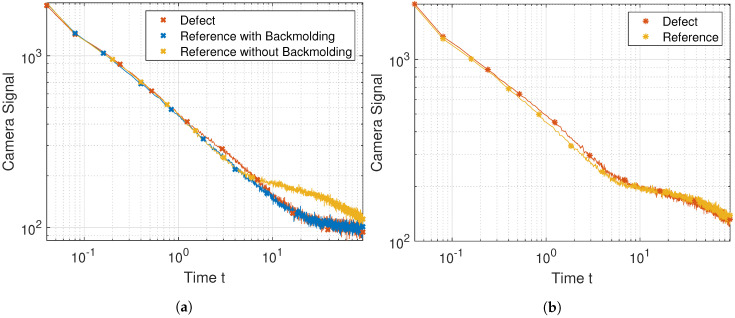
Experimental results: (**a**) measured temperature–time sequences (crosses in Figure 6a) and (**b**) measured temperature–time sequences (asterisks in Figure 6b).

The temperature–time sequences marked as defective in Figure 6a look similar to the simulation data shown in Figure 2a. Certain pixels in Figure 6a are marked by crosses and asterisks, colored red blue and orange. The temperature–time sequences of the crosses are shown in Figure 7a, while those marked with asterisks are shown in Figure 7b. Temperature–time sequences which indicate a defect are colored red.

Figure 7a shows that the temperature buildup caused by the defect was very slight compared to the backmolded reference and that both sequences reached the adiabatic plateau at a similar time. This means that this defect is difficult to find using TSR. The reference without backmolding reached the adiabatic plateau sooner because of the locally reduced sample thickness. This sequence was correctly identified as non-defective by our algorithm.

In Figure 7b we show a defect which was not verifiable by the 3D-XCT measurement. We can see that the defective temperature–time sequence shows a temperature buildup compared to the reference.

**Figure 8:**
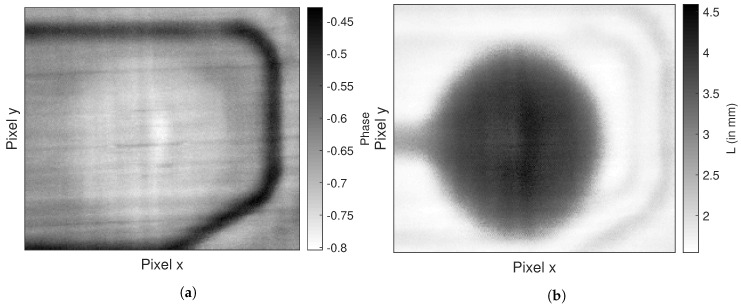
Resultant images of standard evaluation methods of PT: (**a**) PPT and (**b**) TSR.

For comparison, we also show the results of the two most commonly employed algorithms, PPT, in Figure 8a, and TSR, Figure 8b. For PPT, we took the phase of the seventh Fourier-coefficient. This phase image was chosen because it showed the best contrast for the defect found by the 3D-XCT measurement. For TSR we evaluated the measurement using polynomials ranging from n=5 to n=9 and evaluated the largest peak of the second logarithmic derivative. We only show the result of the best defect image which was reached with polynomial degree n=6. We converted the time of the largest peak $t^{*}$ to the specimen thickness *L* using the above mentioned estimate for the effective thermal diffusivty $\alpha_{ez}\;=\;2.3$ ×$10^{-7}$ mm using Equation (5).

In Figure 8a a faint outline of the defect can be seen. In PPT the choice of the Fourier-coefficient is determined by the user. Furthermore, the result of TSR and PPT is merely an image where the defect is not yet segmented. Segmenting the defect would be very difficult without human intervention. In Figure 8b we see that TSR also identifies a faint outline of the defect, albeit much bigger than indicated by the 3D-XCT measurement shown in Figure 6b. Both TSR and our proposed method rely on a polynomial fit, with the main difference being the degree and the evaluation of the roots of the polynomial. For comparison, we show the derivatives of the temperature–time sequences from Figure 7a below. For both methods we show the relevant derivatives, i.e., the second logarithmic derivative for TSR and the first logarithmic derivative of our proposed method.

**Figure 9:**
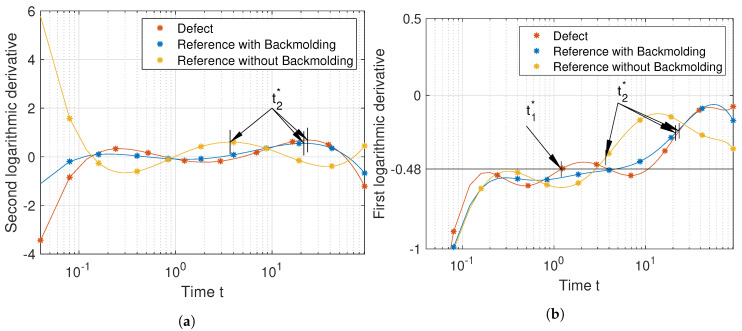
Polynomial derivatives of the temperature–time sequences from Figure 7a: (**a**) second logarithmic derivative of a polynomial of degree *n* = 6 (TSR method) and (**b**) the first logarithmic derivative of a polynomial of degree *n* = 9 (proposed method).

In Figure 9a we see that the peak of the second logarithmic derivatives of the defect and the reference with backmolding occur at a similar time. The peaks of the second logarithmic derivative are indicated by the arrows $t_{2}^{*}$. This is the reason for the blurred defect image produced by TSR, Figure 8b. Due to the relatively low degree of the polynomial, it is not possible to identify the interlaminar thermal reflection produced by the defect. Increasing the polynomial does not necessarily lead to a better approximation due to oscillations introduced by the polynomial approximation. In Figure 9b we show the first logarithmic derivative of a polynomial of degree n=9, also using our recommendation for an improved stability of the fit; see Section 3.2. Using our proposed algorithm, we cannot detect $t_{2}^{*}$, which can be found at the half-rise time of the first derivative. However, we are able to detect the line labeled “Defect”, because it shows three intersections with the black line located at −0.48. As discussed in the theoretical part, the first intersection correlates with the defect depth, and the fact that there exist at least three intersections indicates a defect.

## 5. Conclusions

In this paper, we present an extension of the thermographic signal reconstruction method for subsurface defect characterization using optical-excited pulse thermography measurement data. Comparable algorithms require either orders of magnitude more computing power, or lack the detection capability of interlaminar defects in geometrically-complex components. With the standard TSR defects are characterized by a change of the effective thermal diffusivity. However, by directly analyzing the signal characteristics of defects, i.e., the in-between thermal reflections, we can automatically segment defective from sound areas and generate defect maps. These defect maps also provide a defect depth estimate and their computational cost is inexpensive. We can easily implement the proposed method using a simple adaption to the TSR method. We validated our results using real world measurements of a sample from a pre-production run, where a basic assumption of the defects was obtained by 3D X-ray computed tomography. We compared the results of our algorithm to TSR and the pulsed phased thermography and found that our algorithm is very sensitive to defects close to the surface, which might not be structurally integral. Future work could focus on incorporating artificial intelligence, e.g., convolutional neural networks, for further improving the defect detection capabilities based on the features presented in this work.

## Figures and Tables

**Table 1:** Simulation parameters for the numerical solution of the heat equation.

Geometry Units in mm				Material 1, Composite				Material 2, Air		
*D*	*L*	*d*	*w*	$k_{xy}\left[\frac{W}{m\;\cdot \;K}\right]$	$k_{z}\left[\frac{W}{m\;\cdot \;K}\right]$	$\rho [\frac{kg}{m^{3}}]$	$c[\frac{J}{kg\;\cdot \;K}]$	$k_{xyz}\left[\frac{W}{m\;\cdot \;K}\right]$	$\rho [\frac{kg}{m^{3}}]$	$c[\frac{J}{kg\;\cdot \;K}]$
4	3	1	0.2	1.92	0.64	1500	1200	0.0262	1.2	1

## Abbreviations

The following abbreviations are used in this manuscript:

FRP	Fiber reinforced polymer
3D-XCT	3D-Xray computed tomography
UT	Ultrasonic testing
AT	Active Thermography
IR	Infrared
PT	Pulse thermography
PPT	Pulse phase thermography
TSR	Thermographic signal reconstruction
APST	Absolute peak slope time method
DDT	Dynamic thermal tomography
VWC	Virtual wave concept
NDE	Non-destructive evaluation
FBH	Flat bottomed hole
